# Emirati mothers’ perceptions and willingness to serve ultra-processed and traditional foods to young children

**DOI:** 10.3389/fpubh.2026.1960958

**Published:** 2026-10-08

**Authors:** Fatema Saeed Albadi, Alyaa Mohammed Saeed Alsuwaidi, Sana Naser, Meera Omar Aljahoori, Suhail Alhatmi, Kholood Mohamed Almentheri, Ruwaya Aldhaheri, Aysha Fares Aldhaheri, Maryam Almesmari, Ayesha Salem Al Dhaheri, Rubina Sabir, Abdulmannan Fadel, Agathi Argyriadi, Alexandros Argyriadis, Hafiz Muhammad Shahbaz

**Affiliations:** 1 Department of Nutrition and Health, College of Medicine and Health Sciences, United Arab Emirates University, Al Ain, United Arab Emirates; 2 Department of Cognitive Sciences, College of Humanities and Social Sciences, United Arab Emirates University, Al Ain, United Arab Emirates; 3 Department of Psychology, American University in Dubai, Dubai, United Arab Emirates; 4 Department of Nursing, Hellenic Mediterranean University, Heraklion, Greece

**Keywords:** child feeding, cultural familiarity, food choice, maternal perceptions, traditional foods, ultra-processed foods, United Arab Emirates

## Abstract

Early childhood food choices are shaped by caregiver decisions, yet little is known about how Emirati mothers evaluate ultra-processed foods (UPFs) versus traditional foods for children aged 6 months to 5 years. Using a culturally adapted questionnaire, 283 mothers rated 12 foods (6 UPFs, 6 traditional foods) for perceived healthiness, willingness to serve, convenience, tastiness, processing, and cultural familiarity. Traditional foods were rated consistently higher than UPFs for perceived healthiness (3.70 vs. 1.81), willingness to serve (3.60 vs. 2.54), convenience (3.65 vs. 2.26), tastiness (3.83 vs. 3.48), and cultural familiarity (3.89 vs. 2.91), whereas UPFs were rated as more processed (3.14 vs. 2.74) (all *p* < 0.001). Willingness to serve was analyzed using cumulative link mixed models on 3,324 food-level observations from 277 mothers with complete covariate data. In the base model, UPF status was associated with lower willingness to serve (OR = 0.12, 95% CI: 0.04–0.39). In the fully adjusted model, this association was attenuated and was not statistically significant (OR = 1.23, 95% CI: 0.57–2.64), whereas cultural familiarity (OR = 2.35, 95% CI: 2.16–2.55), convenience (OR = 2.28, 95% CI: 2.03–2.56), tastiness (OR = 1.73, 95% CI: 1.59–1.88), and perceived healthiness (OR = 1.64, 95% CI: 1.44–1.85) were positively associated with willingness to serve. Thus, maternal willingness to serve food to young children was more strongly associated with food-specific perceptions than with food type alone, highlighting the importance of culturally familiar and practical alternatives in efforts to reduce early-life reliance on UPFs.

## 1 Introduction

Early childhood is a critical window for growth and development, and it is also the time when lifelong eating habits begin to take shape. Ultra-processed food (UPF) intake patterns established during these years may track from toddlerhood into mid-childhood (1). The home food environment governs much of what children eat, since it determines the range of foods offered to them. Among these foods, UPFs have gained significant research interest in recent years. According to the NOVA classification system, UPFs are industrial formulations made largely from extracted or synthesized ingredients that typically contain additives and are specifically formulated to enhance palatability, convenience, and shelf life (2, 3).

A significant amount of research links high UPF consumption to poor diet quality and adverse health outcomes, including in children and adolescents (4–6). Recent cohort studies have identified family- and individual-level predictors of UPF intake in young children (7–9). These findings indicate that children’s eating habits and caregivers’ decisions about which foods are appropriate jointly shape UPF exposure in early childhood.

However, there is criticism of the use of the NOVA system in food science. Some authors question the consistency and practical interpretation of its categories, particularly in relation to nutrient-dense or functional foods (10). Such classification ambiguity suggests that caregivers’ real-life feeding decisions may rest less on processing category than on their own perceptions of a food; if trained researchers do not always agree on how to classify a given product under NOVA, caregivers making everyday feeding decisions are unlikely to rely on formal processing categories at all, making food-specific perceptions a more ecologically valid focus of study than category membership per se. These concerns are particularly important during infancy and early childhood, when commercially processed products may be introduced early and become normalized within family feeding routines.

The primary gatekeepers of young children’s diets are the parents and caregivers whose feeding decisions are shaped by more than nutrition knowledge alone. Research focused on children’s eating behavior indicates that food provision reflects perceived healthiness, convenience, cultural familiarity, child acceptance, family routines, and parental beliefs about appropriate intake (11–13). Maternal dietary behaviors and beliefs about commercial infant foods further shape children’s early exposure (14, 15). Importantly, convenience remains a significant driver even among caregivers who actively prioritize the healthiness of food (16, 17). Beyond the nutritional value of food, then, maternal knowledge, decision-making in a wider context, and cultural familiarity appear to shape how parents and the public view UPF consumption (18, 19).

This is especially relevant in the context of the United Arab Emirates (UAE), where feeding practices for infants and children are largely shaped by significant changes in social and dietary habits. Research work in the UAE has focused largely on breastfeeding, complementary feeding, and early infant and toddler feeding practices (20–22). Studies have demonstrated that many infants and young children in the UAE do not meet the recommended feeding requirements owing to the early introduction of non-nutritious foods and insufficient dietary diversity (23, 24). Multi-emirate survey and cohort data on complementary feeding in the UAE, none based on a nationally representative probability sample, have documented inadequate dietary quality, yet none of this work has directly assessed exposure to industrially processed, ready-to-eat foods among children under 5 years of age; a recent scoping review of dietary patterns in the UAE explicitly excluded children younger than 4 years because of insufficient evidence for this age group (25), underscoring a genuine and unaddressed gap. This gap is also increasingly recognized at the global policy level: the WHO/UNICEF indicators for assessing infant and young child feeding practices were updated in 2021 to add indicators of unhealthy food and beverage consumption (sweet-beverage consumption, unhealthy food consumption, and zero fruit-or-vegetable consumption) for children 6–23 months (26), and a separate WHO guideline for complementary feeding of infants and young children 6–23 months of age, published in 2023, further recommends that foods high in sugar, salt, or trans fats, and sugar-sweetened beverages, should not be consumed in this age group (27). Consistent with this policy shift, recent UAE cohort data indicate that at 18 months, 65.4% of children had consumed a sentinel unhealthy food and 26.9% had consumed a sugar-sweetened beverage on the previous day, while only 40.4 and 23.1% achieved minimum dietary diversity and a minimum acceptable diet, respectively (22). Although these indicators reflect dietary quality rather than NOVA-defined UPF exposure specifically, they demonstrate that early exposure to nutritionally undesirable foods and beverages is already common among UAE children, reinforcing the relevance of examining UPF-specific feeding decisions in this population.

Against this backdrop, evidence from the wider Middle East and North Africa (MENA) region suggests that traditional dietary patterns in early childhood may be protective. In Lebanon, for example, lower odds of overweight and obesity were reported among preschool children who followed a traditional dietary pattern (28). Despite this context, little is known about how Emirati mothers weigh UPFs against culturally familiar traditional foods when deciding what to serve young children, or which food-level perceptions most strongly drive those decisions.

This study aimed to evaluate the perceptions of Emirati mothers regarding UPFs and traditional foods for children aged 6 months to 5 years using a culturally adapted food-image survey. The study also compared mothers’ ratings of perceived healthiness, willingness to serve, convenience, tastiness, perceived processing, and cultural familiarity for UPFs versus traditional foods. It further examined whether sociodemographic factors, maternal beliefs, feeding practices, and food-level perceptions were associated with willingness to serve these foods. We hypothesized that traditional foods would be perceived more favorably than UPFs and that willingness to serve would be closely related to perceived healthiness, convenience, tastiness, and cultural familiarity.

## 2 Materials and methods

### 2.1 Study design and participants

A cross-sectional food-image survey was used to examine the perceptions of Emirati mothers regarding UPFs and traditional foods for young children in the UAE. Participants were recruited through a convenience sampling approach; the survey link was distributed exclusively through verified United Arab Emirates University-affiliated staff, student, and alumni networks and institutional email distribution lists. No snowball or chain-referral recruitment was used. The study was carried out between November 2025 and March 2026. The target sample size was approximately 250–300 mothers, which was determined based on feasibility, recruitment period, level of access to participants, and the availability of resources; no formal *a priori* power calculation was performed, consistent with the exploratory, hypothesis-generating nature of this cross-sectional survey. The precision of the resulting effect estimates is conveyed by the 95% confidence intervals reported in Tables 1, 2. In total, 283 respondents who self-reported meeting the eligibility criteria completed the survey. The survey was administered entirely online via Google Forms; no paper-based responses were collected.

**Table 1:** Base cumulative link mixed model for predictors of willingness to serve foods.

Variable	OR	95% CI	*p*
Food type (ref: traditional foods)			
UPFs	0.12	0.04–0.39	0.001
Family size (ref: ≤3 children)			
>3 children	1.38	1.10–1.73	0.006
Mother’s age group (ref: 30–39 years)			
≤29 years	1.06	0.84–1.33	0.628
≥40 years	1.06	0.84–1.34	0.628
Education (ref: university degree)			
Secondary or below	1.16	0.93–1.44	0.194
Postgraduate	0.85	0.63–1.15	0.303
Occupation (ref: employed)			
Housewife	1.09	0.90–1.32	0.363
Maternal beliefs			
Read food labels	0.98	0.88–1.09	0.723
Acceptability of fast food (reverse-coded)	0.81	0.73–0.90	<0.001
Child prefers snacks (reverse-coded)	0.91	0.83–0.99	0.036
Feeding practices			
Encourage child to finish food	1.23	1.12–1.34	<0.001
Use snacks to distract child (reverse-coded)	0.90	0.83–0.98	0.017
Record child’s snacks	0.96	0.88–1.05	0.346

OR, odds ratio; CI, confidence interval. Odds ratios are from a cumulative link mixed model for willingness to serve foods (1, never to 5, always), with random intercepts for participant ID and food item. The base model included food type, maternal sociodemographic characteristics, maternal belief items, and feeding-practice items. Reference categories were traditional foods, maternal age 30–39 years, university education, employed status, and ≤ 3 children. For Likert-type predictors, ORs represent the change in odds associated with a one-unit increase in the predictor. The model was based on 3,324 food-level observations from 277 mothers after complete-case handling of missing covariate data. *p* < 0.05 was considered statistically significant. Random effects: *σ*^2^_participant_ = 0.221, *σ*^2^_food_ = 1.133. Marginal *R*^2^ = 0.224; conditional *R*^2^ = 0.450.

**Table 2:** Extended cumulative link mixed model for predictors of willingness to serve foods, including food-specific perception factors.

Variable	OR	95% CI	*p*
Food type (ref: traditional foods)			
UPFs	1.23	0.57–2.64	0.593
Food-specific perception factors			
Perceived healthiness	1.64	1.44–1.85	< 0.001
Perceived convenience	2.28	2.03–2.56	< 0.001
Perceived tastiness	1.73	1.59–1.88	< 0.001
Perceived processing	1.01	0.96–1.07	0.732
Cultural familiarity	2.35	2.16–2.55	< 0.001
Family size (ref: ≤3 children)			
>3 children	1.44	1.10–1.87	0.007
Mother’s age group (ref: 30–39 years)			
≤29 years	1.03	0.79–1.34	0.825
≥40 years	1.01	0.77–1.32	0.934
Education (ref: university degree)			
Secondary or below	1.26	0.98–1.62	0.076
Postgraduate	1.15	0.80–1.64	0.450
Occupation (ref: employed)			
Housewife	1.07	0.86–1.33	0.553
Maternal beliefs			
Read food labels	1.01	0.89–1.14	0.857
Acceptability of fast food (reverse-coded)	0.97	0.87–1.10	0.673
Child prefers snacks (reverse-coded)	0.92	0.83–1.02	0.110
Feeding practices			
Encourage child to finish food	1.13	1.02–1.26	0.024
Use snacks to distract child (reverse-coded)	0.95	0.86–1.05	0.352
Record child’s snacks	1.00	0.90–1.10	0.968

OR, odds ratio; CI, confidence interval. The extended model included all covariates in Table 1 plus food-specific perception ratings (perceived healthiness, convenience, tastiness, perceived processing, and cultural familiarity), with random intercepts for participant ID and food item. Reference categories were traditional foods, maternal age 30–39 years, university education, employed status, and ≤ 3 children. For Likert-type predictors, ORs represent the change in odds associated with a one-unit increase in the predictor. The model was based on 3,324 food-level observations from 277 mothers after complete-case handling of missing covariate data. *p* < 0.05 was considered statistically significant. Random effects: *σ*^2^_participant_ = 0.364, *σ*^2^_food_ = 0.406. Marginal *R*^2^ = 0.604; conditional *R*^2^ = 0.679.

Eligibility criteria included Emirati mothers, 18 years of age or older, who had at least one child aged 6 months to 5 years and were residing in the UAE. Mothers who had more than one child within the eligible age range were instructed to respond with reference to their youngest child in that range, who served as the index child for the food-evaluation task. Exclusion criteria were mothers whose children had major feeding disorders or long-term illnesses, mothers enrolled in another food or nutrition study, and mothers who could not complete the survey because of time constraints. Eligibility was not enforced through automated skip logic; rather, it was addressed through targeted recruitment (distribution exclusively via UAEU-affiliated networks explicitly describing the target population of Emirati mothers) and through *post hoc* plausibility checks using the sociodemographic items collected (e.g., emirate of residence; age of the youngest child, noting that this item refers to the mother’s youngest child overall and, as described in Section 2.3, does not always confirm the age of the specific index child rated in the food-evaluation task), together with exclusion of illogical or internally inconsistent entries as described below. This self-report, non-gated approach cannot fully rule out a small number of ineligible respondents, which we note as a limitation in Section 4. Ethical clearance was granted by the UAEU Social Sciences Ethics Committee-Research/Course, United Arab Emirates University (Approval No. ERSC_2025_7384). On the participant information page, participants were informed that involvement was voluntary, anonymous, and confidential; that respondents could skip any question; and that proceeding with the questionnaire indicated consent. In the current analysis, no personal identifying data were included. To minimize the risk of automated bot responses, the survey was distributed via verified university networks and email lists, and responses with illogical entries or improbable completion times were excluded prior to analysis.

### 2.2 Survey development and study instruments

Mothers’ evaluation of foods for their young children was examined using a culturally adapted food-image approach. The questionnaire (Supplementary File S4) had two main sections. The first section addressed sociodemographic data, and the second section consisted of a food-photograph evaluation task that included 6 UPFs and 6 traditional/non-UPF food items that are frequently used in the UAE eating environment. Classification of foods was based on the NOVA system (2, 3). Before the rating task, mothers were given a brief written definition of UPFs (foods that are industrially produced and contain added sugars, fats, salt, flavorings, and preservatives, with packaged snacks, sweetened beverages, instant noodles, and processed breakfast cereals given as examples) to orient them to the concept. Individual food images were not labeled by processing category, however, and mothers rated each image solely on the basis of the photograph; food-type classification (UPF vs. traditional) for analysis was assigned by the research team according to the NOVA system and was not disclosed to participants. Mothers were asked to rate each food image using 5-point Likert-type scales for perceived healthiness, willingness to serve, convenience, tastiness, perceived processing level, and cultural familiarity. Mothers were instructed that if they had more than one child in the eligible age range, they should answer with their youngest eligible child in mind.

Food images were gathered from two sources. Standardized food photographs were selected from A Photographic Atlas of Food Portions for the Emirate of Abu Dhabi (29). Additional images of selected home-prepared foods that were not covered by the atlas were generated by the research team. Each image was checked for recognizability, cultural fit, framing, and overall comparability (30). Most food images depicted plated, ready-to-eat dishes without visible commercial packaging; however, three of the six UPF images (packaged snack cake with juice box, sugar-sweetened beverages, and chocolate bar with packaged cookies) and one of the six traditional-food images (laban, a commercially bottled dairy drink) showed visible product packaging or branding, reflecting how these items are typically encountered in the UAE market. This heterogeneity in presentation format is addressed as a limitation in Section 4. The traditional/non-UPF category was defined at the level of the specific dish depicted for this study, using the available ingredient or product description together with NOVA criteria, and does not certify that every ingredient in every household or commercially prepared version of a given dish was non-ultra-processed (i.e., NOVA groups 1–3); complete standardized recipes were not available for all items, so the possibility that some home-prepared or commercially available versions of certain composite traditional dishes contain a NOVA group 4 ingredient cannot be excluded. This classification uncertainty is discussed further in Section 4.

The survey was designed based on existing studies of maternal food-offering choices and child feeding practices, especially the framework used by Vidal et al. (16), along with prior research on parental dietary behaviors and maternal food-offering decisions (11, 13). As specified in the approved study protocol, the survey was developed by adapting context-based questions, translating into Arabic and back-translating into English, and using visual stimuli to confirm recognition and cultural relevance. A pilot study was conducted involving 10–15 participants to assess the clarity, length, cultural sensitivity, and technical feasibility of the survey.

### 2.3 Study variables

Maternal willingness to serve each food to her index child was represented as an ordered outcome with scores ranging from 1 to 5 and was considered the primary outcome for inferential analysis. Food type, classified as UPF or traditional/non-UPF food, was the primary predictor at the food level. Additional variables based on food-level perception included perceived healthiness, convenience, tastiness, perceived processing, and cultural familiarity. Higher scores reflected greater perception of the respective variables.

Participant-level covariates included maternal age group, emirate of residence, education, occupation, family size, and age of the youngest child. The recorded age of the youngest child refers to the mother’s youngest child overall and may therefore fall below the eligible age range; it does not necessarily correspond to the index child used for the food-evaluation task, and the exact age of the index child was not separately recorded. Maternal beliefs and feeding practices were assessed using 5-point Likert-type items and entered as individual variables rather than as summed scales. The maternal belief items addressed whether UPFs are harmful to children’s health, whether mothers read food labels, whether they prefer traditional meals over ready-made options, whether it is acceptable to offer fast food occasionally, and whether the child prefers packaged snacks over home-cooked meals. The feeding-practice items addressed control over intake, concern about overweight, encouragement to finish meals, use of snacks to calm or occupy the child, and tracking of the child’s snack intake. Reverse-coded items were re-coded so that higher values reflected more health-conscious beliefs or practices.

### 2.4 Statistical analysis

All statistical analyses were conducted using R (version 4.5.1). Continuous variables were summarized using means and standard deviations (SDs), whereas categorical variables were summarized using frequencies and percentages. For each of the 12 food items, descriptive statistics were generated across the six food-perception dimensions. At the participant level, composite scores were created by averaging each mother’s ratings across the six UPF items and, separately, across the six traditional-food items. These paired within-participant averages were compared using paired t-tests. Because the underlying ratings were ordinal, Wilcoxon signed-rank tests were also conducted as sensitivity analyses to confirm the robustness of the paired-comparison results. Because each mother rated multiple foods, the dataset had a repeated-measures structure. The data were therefore reshaped from wide to long format, yielding 3,396 potential food-level observations (283 participants × 12 foods). Missing values were examined during data preparation. Six mothers had missing information in participant-level covariates, and because each mother contributed 12 food-level observations, complete-case handling removed 72 observations. This left 3,324 observations for multivariable modeling. Thus, mixed-effects models were estimated using 3,324 food-level records from 277 mothers and 12 food items. Given the very small proportion of deleted records relative to the long-format dataset, complete-case analysis was considered acceptable for the primary models.

The main inferential analysis used cumulative link mixed models (CLMMs) with a logit link, treating willingness to serve as an ordered categorical outcome (1–5). Random intercepts were included for both participant ID and food item to account for repeated observations within mothers and clustering across foods. The same random-effect structure (participant and food item) was retained across all models to ensure consistency and to account for clustering.

Food type and the five food-specific perception variables (perceived healthiness, convenience, tastiness, perceived processing, and cultural familiarity) were the primary variables of interest and were included in the multivariable models *a priori*. Candidate participant-level covariates (maternal age group, emirate of residence, education, occupation, family size, and age of youngest child), together with the maternal belief and feeding-practice items, were first screened in unadjusted single-predictor CLMMs (Supplementary Table S3) and were retained if associated with willingness to serve at *p* < 0.25, a conventional screening threshold for multivariable model building (31). For variables with more than two categories, retention was based on a joint likelihood-ratio test across all levels rather than on individual level estimates. Emirate of residence, age of youngest child, two maternal belief items (UPFs are harmful to children’s health; preference for traditional meals over ready-made options), and two feeding-practice items (control over intake; concern about overweight) did not meet this criterion and were not included in the multivariable models. Model 1 (base model) comprised food type and all retained covariates, whereas Model 2 additionally included the five food-specific perception variables. Results were reported as odds ratios (ORs) with 95% confidence intervals (CIs), and *p* < 0.05 was considered statistically significant.

Likert-scale predictor variables were treated as continuous predictors. This is a common analytic approach for 5-point ordered scales when approximate monotonicity and near-linearity are assumed. Generalized variance inflation factors (GVIFs) were examined to assess the effect of collinearity on multivariable models. The adjusted GVIFs did not suggest problematic multicollinearity, as all values were well below the conservative threshold, with a maximum value of 1.99 (equivalent to a maximum standard VIF of 3.96). The proportional odds assumption underlying the CLMMs was assessed in a sensitivity analysis using the equivalent fixed-effects cumulative link model, for which likelihood-ratio tests of nominal effects are available (nominal_test function, ‘ordinal’ package in R). The assumption was not supported for food type, maternal age group, occupation, family size, and four of the five food-specific perception variables (perceived healthiness, convenience, tastiness, and cultural familiarity; all *p* < 0.05), whereas it was supported for education, perceived processing, and all maternal belief and feeding-practice items (all *p* > 0.05). In the partial proportional-odds model, which allowed threshold-specific (nominal) effects for the violating predictors, perceived healthiness, convenience, tastiness, and cultural familiarity retained positive associations across all response thresholds, although their magnitudes varied; food type and several participant-level covariates showed greater threshold-dependent variation, including estimates that crossed or approached the null. The proportional-odds CLMMs were therefore retained as the primary analysis, and the odds ratios reported in Tables 1, 2 should be interpreted as average effects across the response thresholds. Threshold-specific estimates from this model are reported in Supplementary Table S5. Forest plots were generated using the matplotlib library in Python (version 3.13.5). This study was not pre-registered. The hypotheses and analytic plan were specified prior to data collection and are described in this section.

## 3 Results

### 3.1 Participant characteristics

A total of 283 Emirati mothers were included in the descriptive analyses (Table 3). The mean maternal age was 35.39 ± 8.00 years, with age data available for 277 participants. Half of the participants resided in Abu Dhabi (50.2%), while 18.7% lived in Dubai/Sharjah and 31.1% lived in other emirates. Most participants had a university degree (68.6%), whereas 22.3% had secondary education or below and 9.2% had postgraduate education. A total of 42.8% of participants were housewives and 57.2% were employed/professional. The mean family size was 3.58 ± 2.32 children, with 44.2% of mothers reporting more than three children. The mean age of the youngest child was 8.96 ± 12.17 months, and 65.7% of the youngest children were aged ≤6 months (Table 3).

**Table 3:** Characteristics of the study participants (*N* = 283).

Characteristic	*n*	%
Age of mother (years)^a^ — Mean ± SD = 35.39 ± 8.00		
≤29	81	29.2
30–39	94	33.9
≥40	102	36.8
Emirate of residence		
Abu Dhabi	142	50.2
Dubai/Sharjah	53	18.7
Other^b^	88	31.1
Education level		
Secondary or below	63	22.3
University	194	68.6
Postgraduate	26	9.2
Occupation		
Housewife	121	42.8
Employed/professional	162	57.2
Family size — Mean ± SD = 3.58 ± 2.32		
≤3 children	158	55.8
>3 children	125	44.2
Age of youngest child (months)^c^ — Mean ± SD = 8.96 ± 12.17		
≤6 months	186	65.7
>6 months	97	34.3

Values are *n* (%) unless otherwise indicated.

^a^Age of mother was reported for *n* = 277 (six missing); percentages are based on available data.

^b^Other emirates include Ajman, Fujairah, Ras Al Khaimah, and Umm Al Quwain.

^c^Age of youngest child was reported for all 283 participants; median (IQR) = 5.0 (3.0–10.0) months owing to positive skew. The recorded age refers to the mother’s youngest child overall and may fall below the eligible age range; it does not necessarily correspond to the index child used for the food-evaluation task.

### 3.2 Comparison of ultra-processed and traditional foods

Across the six foods in each category, traditional foods were rated more favorably than UPFs on all evaluative dimensions, while being rated lower on perceived processing (Table 4). Compared with UPFs, traditional foods received significantly higher mean ratings for perceived healthiness (3.70 vs. 1.81), willingness to serve (3.60 vs. 2.54), convenience (3.65 vs. 2.26), tastiness (3.83 vs. 3.48), and cultural familiarity (3.89 vs. 2.91) (all *p* < 0.001). In contrast, UPFs were rated as significantly more processed than traditional foods (3.14 vs. 2.74, *p* < 0.001). Excluding the willingness-to-serve outcome, the largest standardized differences among the perception dimensions were for perceived healthiness (*d* = −3.58, 95% CI: −3.89 to −3.26), convenience (*d* = −1.75, 95% CI: −1.93 to −1.56), and cultural familiarity (*d* = −1.09, 95% CI: −1.24 to −0.94), indicating that the contrast between food categories was substantial in magnitude, not merely statistically significant (Table 4).

**Table 4:** Comparison of ultra-processed and traditional foods across perception dimensions (*N* = 283).

Dimension	UPFs M (SD)	Traditional M (SD)	Mean diff.	*t*	Cohen’s *d* (95% CI)	*p*
Perceived healthiness	1.81 (0.48)	3.70 (0.24)	−1.889	−60.23	−3.58 (−3.89, −3.26)	<0.001
Willingness to serve	2.54 (0.66)	3.60 (0.60)	−1.058	−19.29	−1.15 (−1.29, −0.99)	<0.001
Perceived convenience	2.26 (0.67)	3.65 (0.34)	−1.389	−29.38	−1.75 (−1.93, −1.56)	<0.001
Perceived tastiness	3.48 (0.82)	3.83 (0.55)	−0.360	−6.00	−0.36 (−0.48, −0.24)	<0.001
Perceived processing	3.14 (1.04)	2.74 (1.04)	+0.392	4.01	0.24 (0.12, 0.36)	<0.001
Cultural familiarity	2.91 (0.72)	3.89 (0.61)	−0.972	−18.42	−1.09 (−1.24, −0.94)	<0.001

Values are participant-level mean ratings averaged across six UPFs and six traditional foods. M, mean; SD, standard deviation; *t*, paired-samples *t* statistic; *d*, Cohen’s *d* for paired samples (dz = *t*/√*n*); CI, confidence interval. Mean differences were calculated as UPFs minus traditional foods; negative values indicate higher scores for traditional foods. All scales range from 1 to 5.

A similar general pattern was confirmed by the non-parametric sensitivity analysis. Despite the ordinal structure of the initial ratings, Wilcoxon signed-rank tests showed significant differences between UPFs and traditional foods for the examined parameters (all *p* < 0.001), indicating the robustness of the paired-comparison findings (Supplementary Table S1).

### 3.3 Food-specific patterns

Item-level descriptive results showed clear heterogeneity within both food categories (Supplementary Table S2). Among UPFs, instant noodles received the lowest ratings for perceived healthiness (1.35) and willingness to serve (1.92), indicating particularly strong maternal reluctance toward this product. Other UPFs, including sugar-sweetened beverages and chocolate-based snack items, also received low perceived healthiness ratings, although willingness-to-serve scores varied across items. Among traditional foods, laban was one of the most favorably rated items, with high scores for perceived healthiness (3.96), willingness to serve (4.37), and cultural familiarity (4.46). By contrast, sago received comparatively lower ratings for willingness to serve (2.34) and cultural familiarity (2.84) than other traditional foods. These findings indicate that mothers differentiated not only between food categories but also between specific foods within each category (Supplementary Table S2). Unadjusted single-predictor CLMMs examining each predictor domain individually, including food type, sociodemographic characteristics, maternal beliefs, feeding practices, and food-specific perceptions, are presented in Supplementary Table S3. These analyses guided the selection of variables for the multivariable models presented in Tables 1, 2.

### 3.4 Maternal beliefs and feeding practices

Response distributions for maternal beliefs and feeding practices are presented in Tables 5, 6. For maternal beliefs, most mothers selected agreement categories for the statement that UPFs are harmful to children’s health, with 26.5% agreeing and 45.2% strongly agreeing. Agreement was also common for reading food labels when buying snacks for their child (40.6% agree; 22.3% strongly agree) and for preferring traditional meals over ready-made options (37.8% agree; 38.5% strongly agree) (Table 5). For feeding practices, the highest proportion of strong agreement was observed for encouraging the child to finish all food on their plate, with 40.3% agreeing and 30.0% strongly agreeing. Agreement was also common for controlling how much of a particular food the child eats and for concern that the child might become overweight, while fewer mothers agreed with giving snacks just to soothe or occupy the child (Table 6).

**Table 5:** Response frequencies (%) for maternal belief items (*N* = 283).

Belief item	1	2	3	4	5
UPFs are harmful to children’s health	4.6	4.6	19.1	26.5	45.2
Regularly read food labels when buying snacks	0.4	6.4	30.4	40.6	22.3
Prefer cooking traditional meals over ready-made	0.4	6.7	16.6	37.8	38.5
Acceptable to offer fast food occasionally^a^	5.7	11.0	36.7	41.7	4.9
Child prefers packaged snacks over home-cooked^a^	17.3	32.9	27.6	19.1	3.2

Values are percentages of respondents selecting each category. Scale: 1, strongly disagree; 2, disagree; 3, neutral; 4, agree; 5, strongly agree.

^a^Reverse-coded for regression analyses so that higher scores reflect more health-conscious beliefs; frequencies shown here represent the original response categories.

**Table 6:** Response frequencies (%) for maternal feeding-practice items (*N* = 283).

Feeding-practice item	1	2	3	4	5
Control how much my child eats of certain foods	1.1	6.0	18.7	49.1	25.1
Concerned my child will become overweight	3.2	9.9	24.4	35.7	26.9
Encourage my child to finish all food on plate	1.1	9.5	19.1	40.3	30.0
Offer snacks to calm/occupy my child^a^	8.1	18.0	37.5	29.0	7.4
Keep track of snack foods my child eats	11.0	26.1	39.6	17.7	5.7

Values are percentages of respondents selecting each category. Scale: 1, strongly disagree; 2, disagree; 3, neutral; 4, agree; 5, strongly agree.

^a^Reverse-coded for regression analyses so that higher scores reflect more health-conscious practices; frequencies shown here represent the original response categories.

### 3.5 Predictors of willingness to serve foods

Since each participant rated multiple foods, willingness to serve was analyzed using CLMMs with random intercepts for participant ID and food item. Results from the multivariable models are presented in Tables 1, 2 and Figures 1, 2.

**Figure 1:**
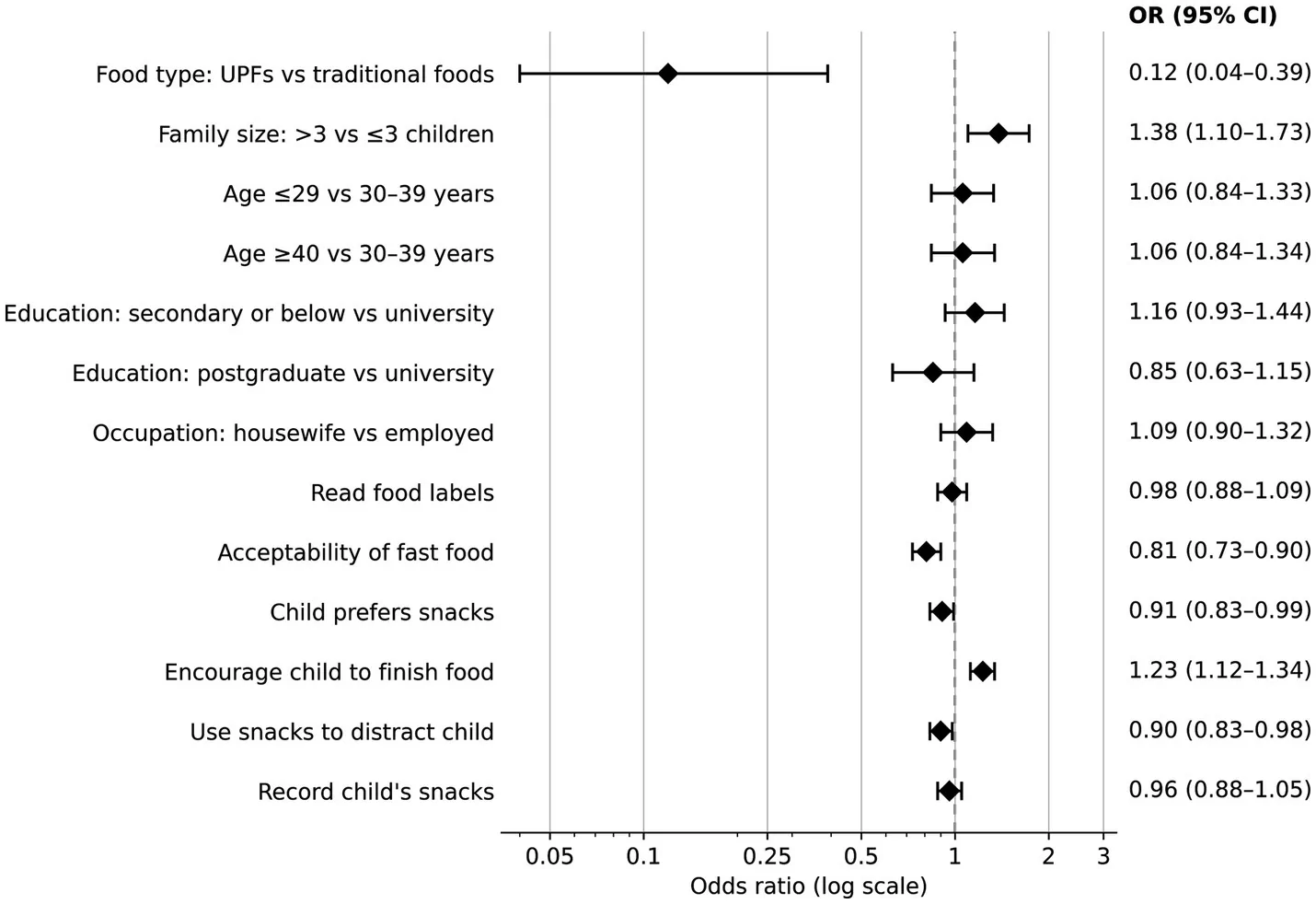
Forest plot of odds ratios (95% confidence intervals) from cumulative link mixed models (CLMMs) for willingness to serve foods: base model without food-specific perception factors. Diamonds represent odds ratios, and horizontal lines indicate 95% confidence intervals. The dashed vertical line represents the null value (OR = 1). Reference categories were traditional foods, maternal age 30–39 years, university education, employed status, and ≤3 children. Confidence intervals that do not cross 1.0 indicate statistical significance (*p* < 0.05).

**Figure 2:**
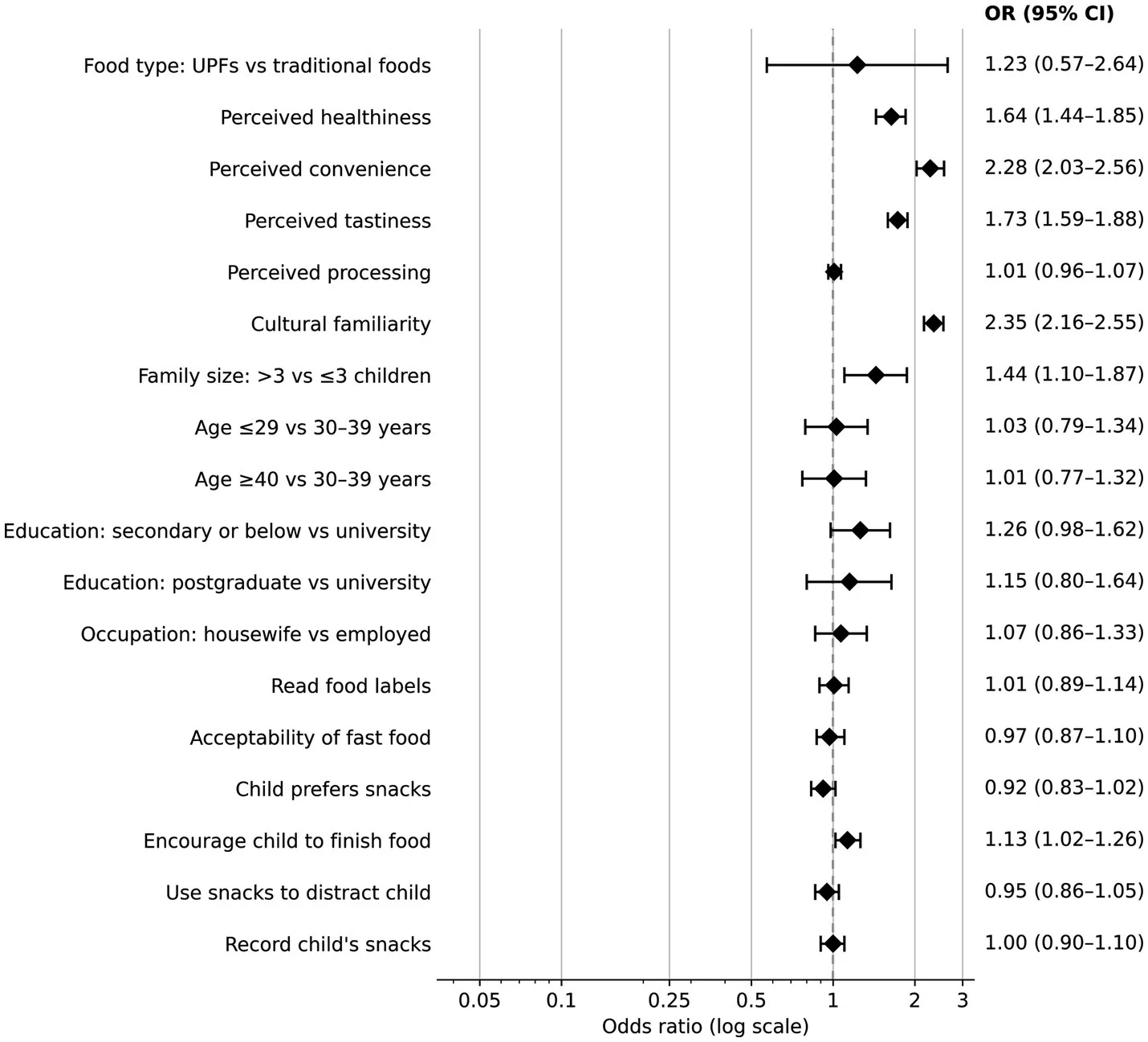
Forest plot of odds ratios (95% confidence intervals) from cumulative link mixed models (CLMMs) for willingness to serve foods: extended model including food-specific perception factors. Diamonds represent odds ratios, and horizontal lines indicate 95% confidence intervals. The dashed vertical line represents the null value (OR = 1). Reference categories were traditional foods, maternal age 30–39 years, university education, employed status, and ≤3 children. Confidence intervals that do not cross 1.0 indicate statistical significance (*p* < 0.05).

#### 3.5.1 Model 1: base model without food-specific perception factors

In the model excluding food-specific perception factors, UPF status was significantly associated with lower willingness to serve (OR = 0.12, 95% CI: 0.04–0.39, *p* = 0.001) (Table 1 and Figure 1). Before accounting for food-level perceptions, mothers had substantially lower odds of reporting a higher willingness-to-serve category for UPFs than for traditional foods. Larger family size (>3 children) was linked to higher willingness overall (OR = 1.38, 95% CI: 1.10–1.73, *p* = 0.006). Among maternal beliefs, higher scores on the reverse-coded acceptability of fast food item (indicating lower acceptance) were associated with lower willingness to serve (OR = 0.81, 95% CI: 0.73–0.90, *p* < 0.001). Similarly, higher scores on the reverse-coded child prefers packaged snacks item (indicating lower preference for packaged snacks) were associated with lower willingness to serve (OR = 0.91, 95% CI: 0.83–0.99, *p* = 0.036). Among feeding practices, encouraging the child to finish food was positively associated with willingness (OR = 1.23, 95% CI: 1.12–1.34, *p* < 0.001), whereas higher scores on the reverse-coded item on using snacks to calm or occupy the child (indicating less frequent use of snacks for this purpose) were associated with lower willingness to serve (OR = 0.90, 95% CI: 0.83–0.98, *p* = 0.017). Maternal age, education, occupation, reading food labels, and recording snacks were not significant in this base model (Table 1 and Figure 1).

The random-effects structure indicated that clustering by food item was stronger than clustering by participant in this model. Food-item variance was 1.133, compared with a participant-level variance of 0.221. The marginal *R*^2^ was 0.224, and the conditional R^2^ increased to 0.450 after inclusion of random effects (Table 1).

#### 3.5.2 Model 2: extended model including food-specific perception factors

After the addition of food-specific perception factors, the association between food type and willingness to serve was attenuated and no longer statistically significant (OR = 1.23, 95% CI: 0.57–2.64, *p* = 0.593) (Table 2 and Figure 2). Instead, four food-level perception variables were independently associated with higher willingness to serve. Cultural familiarity was the strongest correlate (OR = 2.35, 95% CI: 2.16–2.55, *p* < 0.001), followed by convenience (OR = 2.28, 95% CI: 2.03–2.56, *p* < 0.001). Tastiness (OR = 1.73, 95% CI: 1.59–1.88, *p* < 0.001) and perceived healthiness (OR = 1.64, 95% CI: 1.44–1.85, *p* < 0.001) were also positively associated with willingness to serve. By contrast, perceived processing was not independently associated with willingness to serve (OR = 1.01, 95% CI: 0.96–1.07, *p* = 0.732) (Table 2 and Figure 2).

Among participant-level variables in the fully adjusted model, family size >3 children remained significantly associated with higher willingness to serve (OR = 1.44, 95% CI: 1.10–1.87, *p* = 0.007). Encouraging the child to finish all food also remained significant, although with a smaller effect than in the base model (OR = 1.13, 95% CI: 1.02–1.26, *p* = 0.024). None of the maternal belief variables remained independently significant after perceptual factors were included, and maternal age, education, occupation, snack distraction, and snack recording were also non-significant (Table 2 and Figure 2).

The random-effects variances in the fully adjusted model were 0.364 for participant ID and 0.406 for food item, indicating more balanced clustering after inclusion of item-specific ratings. Model fit improved after the inclusion of food-specific perception ratings, with the marginal *R*^2^ increasing to 0.604 and the conditional *R*^2^ to 0.679. Together, the two models showed that the association between food type and willingness to serve was attenuated after adjustment for perceptual factors, while higher perceived cultural familiarity, convenience, tastiness, and healthiness were each associated with greater willingness to serve (Tables 1, 2 and Figures 1, 2).

## 4 Discussion

This study evaluated how Emirati mothers assessed UPFs and traditional foods when deciding what they were willing to serve to young children. The study has two primary findings. First, traditional foods received higher ratings than UPFs for perceived healthiness, willingness to serve, convenience, tastiness, and cultural familiarity. Second, the association between food type and willingness to serve was attenuated after adjustment for food-level perception ratings. Cultural familiarity, convenience, tastiness, and perceived healthiness were positively associated with willingness to serve in the fully adjusted model. These results indicate that mothers’ willingness to serve foods was closely related to food-specific perceptions in practical and cultural terms, in addition to food category. Understanding the perceptual factors influencing mothers’ willingness to serve food is especially important for early childhood nutrition in the UAE, as it has been demonstrated that many young children in the UAE do not meet nutrient recommendations (23). To our knowledge, this is the first study in the UAE, and one of few in the wider Gulf and MENA region, to quantify, using a repeated-measures, mixed-effects framework, whether maternal willingness to serve foods to young children tracks more closely with formal processing category (UPF vs. traditional) or with food-specific perceptual attributes such as cultural familiarity and convenience. This distinction is not merely descriptive: it indicates whether processing-category messaging or perception-based communication (e.g., emphasizing convenience and cultural familiarity) is more strongly associated with mothers’ willingness to serve, a question prior work in this field (16, 18, 19) has raised; because no intervention was tested; because no intervention was tested, our cross-sectional design cannot establish which approach would be more effective at changing feeding behavior in practice.

The more favorable evaluation of traditional foods was consistent with a broad literature showing that higher UPF consumption was associated with poorer diet quality and adverse health outcomes across the life course (4, 5), including in children and adolescents (6).

Although the first 1,000 days of life represent a critical window for shaping these early exposures (32), longitudinal data on early exposure to UPFs during infancy and toddlerhood are scarce. According to research from Brazil, UPF intake is linked to maternal, family, and household variables and occurs early in childhood, including before the age of two (33, 34). In the present study, mothers rated UPFs as less healthy and showed lower willingness to serve them in unadjusted comparisons, despite the fact that UPFs are consumed at an early age. This pattern is broadly consistent with the wider literature on a possible divergence between stated attitudes and actual dietary exposure; however, because the exposure estimates cited above come from different populations (Brazilian cohorts) rather than from our own sample, our data cannot directly establish such a gap within this cohort, and this comparison should be read as suggestive rather than as within-sample evidence of an attitude-exposure discrepancy.

The mixed-model results provide a more precise interpretation than comparing categories alone. In the base model, UPFs were strongly linked to lower willingness to serve, but once perceptions of the foods were taken into account, that association weakened and became statistically non-significant. Because food type, food-specific perceptions, and willingness to serve were all measured concurrently within the same cross-sectional survey, this attenuation cannot be interpreted as evidence of mediation or of a causal pathway; establishing mediation would require temporal separation between exposure, mediator, and outcome, which this design does not provide. Descriptively, however, the pattern indicates that food type and food-specific perceptions share a substantial portion of overlapping association with willingness to serve, such that once perceptions are accounted for, food type alone adds little further explanatory value in this sample. The base model captures the overall association of food type with willingness to serve, while the second shows what remains after accounting for those perceptual impressions. The attenuation therefore does not imply that processing is irrelevant; it suggests instead that a large part of the association between food type and willingness to serve overlaps with how these foods are viewed culturally and practically, consistent with the non-causal, descriptive framing above.

This is consistent with findings from a study conducted in Uruguay, where mothers’ decisions about whether to include UPFs during early childhood were shaped not only by the foods themselves but by a combination of beliefs about processing, nutrition, and everyday feeding demands (16). In earlier work, Vidal et al. (35) found that parents’ food choices in early childhood rely on a wider web of aims, routines, and situational pressures rather than being driven solely by nutrition knowledge. The findings of the present study extend this idea by quantifying associations between food-level perceptions and willingness to serve, using a repeated-measures comparison of UPFs and traditional foods. Of all the perceptual factors examined, cultural familiarity emerged as the strongest correlate of willingness to serve in the full model. When mothers evaluated a food, therefore, the decision was not based on healthiness alone, but also on whether the food represented a familiar and natural fit for the everyday family table. Mothers’ own ways of sorting and classifying foods are also likely to steer daily choices (36). In the Middle Eastern context this is highly relevant, because traditional foods are deeply embedded in the cultural fabric and carry historical weight and social meaning, which makes them an easier and more natural choice for everyday meals (37).

The findings of the present study are in agreement with emerging research on public and caregiver perceptions of UPFs. In a recent narrative review, McCausland et al. (19) reported that knowledge, interpretive context, and cultural familiarity shape public and expert views regarding UPFs. Girona et al. (18) reported that parental decisions regarding UPFs are influenced by social, family, and environmental realities rather than being based purely on nutritional judgments. Our findings also align with regional evidence suggesting that traditional dietary patterns may carry child-health advantages. For example, a study conducted in Lebanon reported lower odds of overweight and obesity among preschool children who followed a traditional dietary pattern (28). Although diet quality and child adiposity were not measured in the present study, the strong positive association between cultural familiarity and willingness to serve suggests that traditional foods may carry both cultural and practical value in early child-feeding decisions.

Attention should be given to the role of convenience in dietary choices. In the international literature, convenience is a common driver in the selection of commercial infant foods and UPFs for young children by caregivers (16–18, 38). At the same time, lower intake of UPFs by children has been associated with higher parental cooking confidence, suggesting that household food skills may shape whether convenience is sought through packaged products or home-prepared options (39). However, in the present study, traditional foods were rated as more convenient overall than UPFs, and convenience remained one of the strongest correlates of willingness to serve in the fully adjusted model. This suggests that convenience is context-dependent rather than inherently tied to industrially processed products. In the present sample, convenience may have reflected routine household preparation, cultural normalization, or familiarity with traditional foods as acceptable child-feeding options. This indicates that traditional foods were not viewed as less practical than UPFs. Notably, one traditional item in our stimulus set (laban) is itself commercially packaged and sold ready-to-serve in the UAE, illustrating that ready-made commercial availability is not unique to UPFs and may itself contribute to perceived convenience independently of processing category. However, the survey captured only mothers’ perceptions of the depicted food stimuli; it did not establish whether a given traditional food was imagined as home-prepared, purchased ready-made, or prepared by another household member, nor did it measure preparation time, cost, or availability. Convenience ratings should therefore be interpreted as perceptions of the presented foods rather than as objective measures of preparation burden; this distinction is addressed further in the limitations below.

A greater willingness to serve was independently associated with perceived tastiness as well as perceived healthiness, which shows that both sensory appeal and nutritional value play an important role in food decisions. These findings are consistent with earlier research indicating that parents consider their child’s food preferences and their own beliefs about healthfulness when deciding which foods to offer (11, 12). In this study, perceived tastiness showed a strong association with willingness to serve. This indicates that mothers’ readiness to offer foods was associated not only with how healthy the foods appeared but also with whether the foods were expected to be appealing to children.

In the fully adjusted model, greater willingness to serve was also linked to larger family size and to encouraging the child to finish the food. This suggests that mothers’ food choices for their children may be shaped by household structure as well as by routine feeding practices. Previous studies have highlighted the importance of the home feeding environment in children’s exposure to UPFs and have also shown associations between parental feeding practices and UPF consumption (40, 41). Moreover, evidence from a UK cohort indicates that UPF intake patterns established in toddlerhood can persist into mid-childhood (1), consistent with the tracking pattern noted in the Introduction. Research in preschool populations has identified child- and family-level predictors that influence food choices (42). Whether the association between family size and willingness to serve is itself modified by food-specific perceptions such as convenience (e.g., whether mothers of larger families rate UPFs as particularly convenient) was not tested in our *a priori* analytic plan and would require a dedicated interaction model; we flag this as a specific direction for future analysis rather than introducing an unplanned *post hoc* interaction term, which would raise multiple-comparison concerns given the number of covariates already included in Table 2. Our findings are therefore consistent with the broader literature, suggesting that mothers’ willingness to serve particular foods during early childhood is shaped not only by food-specific judgments but also by the wider context of household feeding routines.

In the fully adjusted model, perceived processing was not independently linked to willingness to serve. Descriptively, mothers viewed UPFs as more processed, but once other variables entered the model, processing did not independently correlate with willingness. Processing was therefore not a standalone factor in decision-making; it operated in combination with the broader evaluations that caregivers make in real-time feeding decisions. This is relevant to the ongoing debate regarding the NOVA classification system. Monteiro et al. (3) remain foundational for defining UPFs, yet Braesco et al. (10) have raised concerns about the system’s operational clarity, stating that its criteria for identifying UPFs are not always clearly operationalized and can lead to inconsistent classification across assessors and data sources. More recently, Monteiro et al. (43) reaffirmed the public health relevance of the UPF classification concept, even as food systems continue to shift. These findings do not challenge the utility of NOVA for classifying foods by processing level; rather, they suggest that caregivers’ willingness to serve foods may depend more strongly on perceived healthiness, familiarity, convenience, and taste than on perceived processing alone.

The findings of the present investigation are relevant to the UAE setting. Previous studies in the UAE have mainly focused on breastfeeding, complementary feeding, and infant and toddler feeding practices (20, 21) and have reported inconsistencies in breastfeeding practices and early weaning, which are contrary to recommended feeding guidelines. The results of the present study add a complementary perspective to the existing literature by highlighting how mothers assess specific foods once children shift into the wider family food environment. Despite the changing food environment, this study showed that traditional foods retain a perceptual advantage in determining food choices.

In practice, these findings suggest that efforts to reduce reliance on UPFs should not focus on simple avoidance messages alone. Even when caregivers recognize the health concerns associated with UPFs, these foods can still be regarded as acceptable if they are familiar and convenient and fit seamlessly into existing household routines. Interventions might therefore be more effective if the focus is placed on making healthier traditional foods more accessible, convenient, and easy to incorporate into daily routines, rather than relying solely on broad warnings about processing.

This study has several strengths. It addresses a key evidence gap in the UAE by directly weighing UPFs against traditional foods. Methodologically, it employed CLMMs, which handled the ordinal nature of the willingness-to-serve ratings and the clustered structure of repeated observations. However, there are several limitations to this work. The cross-sectional study design prevents firm causal conclusions from being drawn. A non-probability sampling method was adopted that might not fully represent all Emirati mothers: recruitment through university-affiliated networks likely over-represents mothers with higher educational attainment, greater digital access, or a stronger interest in food, cooking, or child nutrition than the general Emirati population, which could bias perception ratings toward more health-conscious and culturally favorable responses; household income was not collected in this study and could therefore not be evaluated as a potential source of selection bias. Eligibility (Emirati nationality, UAE residency, and having a child in the target age range) was assessed through targeted recruitment and self-reported questionnaire information rather than through automated skip logic; because no direct identifying information was collected, respondents’ nationality, maternal status, and residency could not be independently authenticated, and a small number of ineligible responses cannot be entirely ruled out, although illogical or internally inconsistent entries were removed before analysis. The food images also varied in presentation format: a subset of items, predominantly packaged snack and beverage products and the traditional item laban, displayed visible commercial packaging or branding, whereas the remaining items were shown as plated, home-style dishes; this heterogeneity may have contributed to ratings of convenience and perceived processing independently of the food’s NOVA category, and future work should standardize image presentation (e.g., uniform plating without visible packaging) to isolate these dimensions more cleanly. The traditional/non-UPF category was also defined at the level of the specific dish depicted for this study rather than validated against every household or commercially prepared version of that dish; because complete standardized recipes were not available for all items, some home-prepared or commercially available versions of certain composite traditional dishes may contain a NOVA group 4 ingredient, and this classification uncertainty cannot be entirely excluded. Similarly, convenience ratings captured mothers’ perceptions of the depicted food stimuli but did not distinguish whether a food was imagined as home-prepared, purchased ready-made, or prepared by another household member, nor did the survey measure preparation time, cost, or availability; convenience ratings should therefore be interpreted as perceptions of the presented foods rather than as objective measures of preparation burden. Providing participants with a general definition of UPFs before the rating task, while necessary to orient them to the study construct, may also have introduced a degree of demand characteristics that could not be fully separated from genuine perceptual judgments. In addition, the proportional-odds assumption was not supported for several predictors in sensitivity testing, so the odds ratios reported here should be read as average effects across response thresholds rather than as constant effects at every threshold. Because that sensitivity model was a fixed-effects model that did not incorporate the participant- and food-level random intercepts used in the primary models, its threshold-specific estimates should be regarded as illustrative and are not directly comparable in magnitude with the primary estimates. These points may be addressed through future prospective cohort or longitudinal study designs, which would allow the temporal and causal inferences that the current cross-sectional, observational design cannot support.

## 5 Conclusion

Emirati mothers rated traditional foods more favorably than UPFs in five domains: perceived healthiness, willingness to serve, convenience, tastiness, and cultural familiarity, whereas UPFs were perceived as more processed. In the base model, UPF status was associated with lower willingness to serve, but this association was attenuated and no longer statistically significant after adjustment for food-specific perception ratings. In the extended model, cultural familiarity, convenience, tastiness, and healthiness were positively associated with willingness to serve. These findings suggest that maternal willingness to serve foods to young children was closely related to how foods were perceived in practical, sensory, and cultural terms. The findings also indicate that efforts to reduce early-life reliance on UPFs may benefit from supporting healthier foods that are culturally familiar, acceptable, and practical for routine family feeding.

## Data Availability

The datasets presented in this article are not readily available because ethical restrictions relating to participant confidentiality apply. De-identified data may be made available by the corresponding author upon reasonable request and subject to approval by the UAEU Social Sciences Ethics Committee. Requests to access the datasets should be directed to shahbaz@uaeu.ac.ae.
