# Supplementary material for: Emirati mothers’ perceptions and willingness to serve ultra-processed and traditional foods to young children
Source: Front Public Health. 2026 Oct 8;14:1960958. doi: 10.3389/fpubh.2026.1960958 (PMC13687331; doi:10.3389/fpubh.2026.1960958)
Supplement: Supplementary file 1 [file Supplementary_file_1.docx]

**Supplementary Material**

**Supplementary Table S1.** Wilcoxon signed-rank test results comparing ultra-processed and traditional food ratings across all six perception dimensions (n = 283 mothers). Sensitivity analysis for the paired comparisons reported in Table 4 of the main article.

| **Dimension** | **W statistic** | **Hodges–Lehmann estimate**^a^ | **95% CI** | **p** |
| --- | --- | --- | --- | --- |
| Perceived healthiness | 0.0 | −1.917 | (−2.000, −1.833) | < 0.001 |
| Willingness to serve | 2256.5 | −1.167 | (−1.250, −1.000) | < 0.001 |
| Perceived convenience | 385.5 | −1.417 | (−1.583, −1.333) | < 0.001 |
| Perceived tastiness | 9796.0 | −0.417 | (−0.500, −0.250) | < 0.001 |
| Perceived processing | 23348.5 | 0.333 | (0.167, 0.583) | < 0.001 |
| Cultural familiarity | 2324.0 | −1.000 | (−1.083, −0.917) | < 0.001 |

CI, confidence interval. Comparisons are within-participant, based on each mother's mean rating across the six ultra-processed food items versus her mean rating across the six traditional food items. All scales range from 1 to 5.

^a^Estimates are expressed as ultra-processed minus traditional foods. Negative estimates indicate higher ratings for traditional foods; positive estimates indicate higher ratings for ultra-processed foods.

**Supplementary Table S2.** Item-level descriptive statistics for each of the 12 food items across the six perception dimensions (N = 283 mothers).

| **Type** | **Food item** | **Dimension** | **Mean** | **SD** | **Median** | **Skewness** | **Kurtosis** |
| --- | --- | --- | --- | --- | --- | --- | --- |
| UPF | 1. Chicken Nuggets with Fries | Perceived healthiness | 2.03 | 0.75 | 2 | 0.29 | −0.39 |
| UPF | 1. Chicken Nuggets with Fries | Willingness to serve | 2.86 | 0.85 | 3 | 0.14 | 0.26 |
| UPF | 1. Chicken Nuggets with Fries | Perceived convenience | 2.40 | 0.90 | 2 | 0.17 | −0.75 |
| UPF | 1. Chicken Nuggets with Fries | Perceived tastiness | 3.61 | 0.91 | 4 | −0.63 | 0.09 |
| UPF | 1. Chicken Nuggets with Fries | Perceived processing | 3.17 | 1.33 | 3 | −0.35 | −1.05 |
| UPF | 1. Chicken Nuggets with Fries | Cultural familiarity | 3.04 | 1.06 | 3 | 0.14 | −0.65 |
| UPF | 2. Chocolate Cereal with Flavored Milk | Perceived healthiness | 2.25 | 0.89 | 2 | 0.11 | −0.85 |
| UPF | 2. Chocolate Cereal with Flavored Milk | Willingness to serve | 2.59 | 1.04 | 3 | 0.17 | −0.54 |
| UPF | 2. Chocolate Cereal with Flavored Milk | Perceived convenience | 2.54 | 1.04 | 3 | −0.02 | −1.17 |
| UPF | 2. Chocolate Cereal with Flavored Milk | Perceived tastiness | 3.61 | 1.03 | 4 | −0.76 | 0.27 |
| UPF | 2. Chocolate Cereal with Flavored Milk | Perceived processing | 3.17 | 1.25 | 3 | −0.30 | −0.90 |
| UPF | 2. Chocolate Cereal with Flavored Milk | Cultural familiarity | 2.84 | 1.16 | 3 | 0.20 | −0.73 |
| UPF | 3. Packaged Snack Cake + Juice Box | Perceived healthiness | 2.07 | 0.90 | 2 | 0.32 | −0.90 |
| UPF | 3. Packaged Snack Cake + Juice Box | Willingness to serve | 2.75 | 0.98 | 3 | 0.15 | −0.38 |
| UPF | 3. Packaged Snack Cake + Juice Box | Perceived convenience | 2.55 | 0.93 | 3 | −0.11 | −0.86 |
| UPF | 3. Packaged Snack Cake + Juice Box | Perceived tastiness | 3.49 | 1.08 | 4 | −0.55 | −0.22 |
| UPF | 3. Packaged Snack Cake + Juice Box | Perceived processing | 3.19 | 1.23 | 3 | −0.42 | −0.79 |
| UPF | 3. Packaged Snack Cake + Juice Box | Cultural familiarity | 3.00 | 1.06 | 3 | 0.06 | −0.60 |
| UPF | 4. Instant Noodles | Perceived healthiness | 1.35 | 0.64 | 1 | 1.91 | 3.54 |
| UPF | 4. Instant Noodles | Willingness to serve | 1.92 | 0.99 | 2 | 0.94 | 0.27 |
| UPF | 4. Instant Noodles | Perceived convenience | 1.68 | 0.99 | 1 | 1.19 | 0.10 |
| UPF | 4. Instant Noodles | Perceived tastiness | 2.96 | 1.48 | 3 | −0.10 | −1.42 |
| UPF | 4. Instant Noodles | Perceived processing | 2.99 | 1.45 | 4 | −0.44 | −1.49 |
| UPF | 4. Instant Noodles | Cultural familiarity | 2.55 | 1.28 | 2 | 0.46 | −0.92 |
| UPF | 5. Sugar-Sweetened Beverages (SSBs) | Perceived healthiness | 1.57 | 0.82 | 1 | 1.43 | 1.72 |
| UPF | 5. Sugar-Sweetened Beverages (SSBs) | Willingness to serve | 2.57 | 1.10 | 3 | 0.31 | −0.59 |
| UPF | 5. Sugar-Sweetened Beverages (SSBs) | Perceived convenience | 2.12 | 1.07 | 2 | 0.46 | −1.08 |
| UPF | 5. Sugar-Sweetened Beverages (SSBs) | Perceived tastiness | 3.47 | 1.20 | 4 | −0.67 | −0.36 |
| UPF | 5. Sugar-Sweetened Beverages (SSBs) | Perceived processing | 3.14 | 1.33 | 4 | −0.51 | −1.07 |
| UPF | 5. Sugar-Sweetened Beverages (SSBs) | Cultural familiarity | 2.99 | 1.16 | 3 | 0.00 | −0.76 |
| UPF | 6. Chocolate Bar with Packaged Cookies | Perceived healthiness | 1.59 | 0.74 | 1 | 0.86 | −0.50 |
| UPF | 6. Chocolate Bar with Packaged Cookies | Willingness to serve | 2.57 | 0.93 | 3 | 0.09 | −0.25 |
| UPF | 6. Chocolate Bar with Packaged Cookies | Perceived convenience | 2.27 | 0.99 | 2 | 0.15 | −1.10 |
| UPF | 6. Chocolate Bar with Packaged Cookies | Perceived tastiness | 3.72 | 1.22 | 4 | −0.89 | −0.03 |
| UPF | 6. Chocolate Bar with Packaged Cookies | Perceived processing | 3.16 | 1.35 | 4 | −0.42 | −1.11 |
| UPF | 6. Chocolate Bar with Packaged Cookies | Cultural familiarity | 3.03 | 1.15 | 3 | −0.04 | −0.64 |
| Traditional | 1. Grilled Chicken with Rice | Perceived healthiness | 3.96 | 0.20 | 4 | −6.21 | 42.22 |
| Traditional | 1. Grilled Chicken with Rice | Willingness to serve | 4.18 | 0.75 | 4 | −0.70 | 0.52 |
| Traditional | 1. Grilled Chicken with Rice | Perceived convenience | 3.94 | 0.28 | 4 | −4.83 | 24.66 |
| Traditional | 1. Grilled Chicken with Rice | Perceived tastiness | 3.80 | 0.80 | 4 | −0.34 | 0.14 |
| Traditional | 1. Grilled Chicken with Rice | Perceived processing | 2.66 | 1.38 | 3 | 0.13 | −1.40 |
| Traditional | 1. Grilled Chicken with Rice | Cultural familiarity | 4.27 | 0.83 | 4 | −1.15 | 1.30 |
| Traditional | 2. Khubz with Labneh and Dates | Perceived healthiness | 3.92 | 0.31 | 4 | −4.61 | 28.25 |
| Traditional | 2. Khubz with Labneh and Dates | Willingness to serve | 3.28 | 1.14 | 3 | −0.27 | −0.69 |
| Traditional | 2. Khubz with Labneh and Dates | Perceived convenience | 3.66 | 0.70 | 4 | −2.25 | 4.69 |
| Traditional | 2. Khubz with Labneh and Dates | Perceived tastiness | 3.57 | 0.98 | 4 | −0.48 | 0.04 |
| Traditional | 2. Khubz with Labneh and Dates | Perceived processing | 2.70 | 1.36 | 3 | 0.21 | −1.26 |
| Traditional | 2. Khubz with Labneh and Dates | Cultural familiarity | 3.55 | 1.07 | 4 | −0.48 | −0.37 |
| Traditional | 3. Balaleet | Perceived healthiness | 3.59 | 0.60 | 4 | −1.43 | 2.41 |
| Traditional | 3. Balaleet | Willingness to serve | 3.44 | 0.93 | 3 | −0.19 | −0.12 |
| Traditional | 3. Balaleet | Perceived convenience | 3.67 | 0.59 | 4 | −1.90 | 3.84 |
| Traditional | 3. Balaleet | Perceived tastiness | 3.94 | 0.74 | 4 | −0.55 | 0.94 |
| Traditional | 3. Balaleet | Perceived processing | 2.78 | 1.26 | 3 | 0.32 | −0.85 |
| Traditional | 3. Balaleet | Cultural familiarity | 3.98 | 0.99 | 4 | −0.72 | −0.18 |
| Traditional | 4. Vegetable Salona | Perceived healthiness | 3.92 | 0.31 | 4 | −3.93 | 16.05 |
| Traditional | 4. Vegetable Salona | Willingness to serve | 4.00 | 0.98 | 4 | −0.88 | 0.49 |
| Traditional | 4. Vegetable Salona | Perceived convenience | 3.77 | 0.55 | 4 | −2.56 | 6.43 |
| Traditional | 4. Vegetable Salona | Perceived tastiness | 4.00 | 0.85 | 4 | −0.54 | −0.21 |
| Traditional | 4. Vegetable Salona | Perceived processing | 2.55 | 1.47 | 2 | 0.23 | −1.52 |
| Traditional | 4. Vegetable Salona | Cultural familiarity | 4.18 | 0.95 | 4 | −1.09 | 0.69 |
| Traditional | 5. Laban | Perceived healthiness | 3.96 | 0.21 | 4 | −5.82 | 36.95 |
| Traditional | 5. Laban | Willingness to serve | 4.37 | 0.82 | 5 | −1.19 | 0.88 |
| Traditional | 5. Laban | Perceived convenience | 3.94 | 0.28 | 4 | −5.75 | 43.20 |
| Traditional | 5. Laban | Perceived tastiness | 4.27 | 0.78 | 4 | −1.05 | 1.45 |
| Traditional | 5. Laban | Perceived processing | 2.94 | 1.37 | 3 | −0.02 | −1.36 |
| Traditional | 5. Laban | Cultural familiarity | 4.46 | 0.81 | 5 | −1.50 | 1.99 |
| Traditional | 6. Sago | Perceived healthiness | 2.86 | 0.87 | 3 | −0.54 | −0.29 |
| Traditional | 6. Sago | Willingness to serve | 2.34 | 1.00 | 2 | 0.46 | −0.08 |
| Traditional | 6. Sago | Perceived convenience | 2.93 | 0.91 | 3 | −0.68 | −0.22 |
| Traditional | 6. Sago | Perceived tastiness | 3.42 | 1.00 | 4 | −0.53 | 0.08 |
| Traditional | 6. Sago | Perceived processing | 2.82 | 1.43 | 3 | 0.30 | −1.21 |
| Traditional | 6. Sago | Cultural familiarity | 2.84 | 1.20 | 3 | 0.16 | −0.83 |

SD, standard deviation. Higher scores indicate greater perceived healthiness, willingness to serve, perceived convenience, perceived tastiness, perceived processing, and cultural familiarity (all scales 1–5). Food items are numbered within each category and listed in the order used in Supplementary File S4; classification follows the NOVA system.

**Supplementary Table S3.** Unadjusted (single-predictor) cumulative link mixed models for willingness to serve foods, by predictor domain.

Each row reports a separate single-predictor cumulative link mixed model containing only the predictor shown, with random intercepts for participant and food item. Odds ratios (ORs) and 95% confidence intervals (CIs) are therefore unadjusted; p < 0.05 was considered statistically significant. For variables with more than two categories, screening for the multivariable models (Tables 1 and 2 of the main article) was based on a joint likelihood-ratio test across all levels rather than on individual level estimates.

| **Variable** | **OR** | **95% CI** | **p** |
| --- | --- | --- | --- |
| *Food type (ref: traditional foods)* |  |  |  |
| UPFs | 0.11 | 0.03–0.39 | < 0.001 |
| *Food-specific perception factors* |  |  |  |
| Perceived healthiness | 3.09 | 2.78–3.45 | < 0.001 |
| Perceived convenience | 4.21 | 3.81–4.65 | < 0.001 |
| Perceived tastiness | 2.64 | 2.44–2.85 | < 0.001 |
| Perceived processing | 1.06 | 1.01–1.11 | 0.025 |
| Cultural familiarity | 3.24 | 2.99–3.50 | < 0.001 |
| *Family size (ref: ≤ 3 children)* |  |  |  |
| > 3 children | 1.47 | 1.21–1.78 | < 0.001 |
| *Mother's age group (ref: 30–39 years)* |  |  |  |
| ≤ 29 years | 0.94 | 0.74–1.20 | 0.636 |
| ≥ 40 years | 1.24 | 0.98–1.56 | 0.072 |
| *Education (ref: university degree)* |  |  |  |
| Secondary or below | 1.30 | 1.03–1.65 | 0.030 |
| Postgraduate | 0.79 | 0.57–1.12 | 0.185 |
| *Occupation (ref: employed)* |  |  |  |
| Housewife | 1.26 | 1.03–1.53 | 0.023 |
| *Emirate of residence (ref: Abu Dhabi)* |  |  |  |
| Dubai/Sharjah | 0.89 | 0.69–1.16 | 0.395 |
| Other emirates | 1.15 | 0.92–1.44 | 0.219 |
| *Age of youngest child (ref: > 6 months)* |  |  |  |
| ≤ 6 months | 1.03 | 0.84–1.27 | 0.778 |
| *Maternal beliefs* |  |  |  |
| UPFs harmful to health | 0.97 | 0.89–1.06 | 0.477 |
| Read food labels | 0.88 | 0.78–0.98 | 0.020 |
| Prefer traditional meals | 0.99 | 0.89–1.10 | 0.833 |
| Acceptability of fast food | 0.76 | 0.69–0.84 | < 0.001 |
| Child prefers snacks | 0.83 | 0.76–0.91 | < 0.001 |
| *Feeding practices* |  |  |  |
| Control child's intake | 1.06 | 0.94–1.18 | 0.335 |
| Worry about child overweight | 0.99 | 0.90–1.08 | 0.809 |
| Encourage child to finish food | 1.26 | 1.15–1.39 | < 0.001 |
| Use snacks to distract child | 0.83 | 0.75–0.91 | < 0.001 |
| Record child's snacks | 0.94 | 0.86–1.04 | 0.216 |

OR, odds ratio; CI, confidence interval; UPF, ultra-processed food. Models were fitted on 3,324 food-level observations from 277 mothers after complete-case handling of missing covariate data. Maternal belief and feeding-practice items were entered as continuous 5-point predictors, with reverse-coded items re-coded so that higher values reflect more health-conscious beliefs or practices.

**Supplementary File S4.** Complete survey instrument (English version): Mother and Child Food Perception Questionnaire, comprising the participant information and consent text, demographic questions, the food-image evaluation task, and the food stimulus list.

*Culturally adapted food-image questionnaire administered to Emirati mothers. The instrument was prepared in English and Arabic, with independent back-translation, and is reproduced here in its English version.*

## Participant information and consent

This questionnaire is part of the study “Why Emirati Mothers Choose Ultra-Processed Foods for Young Children: A Mixed-Methods Study of Cultural and Perceptual Drivers in the UAE.” It aims to explore how Emirati mothers perceive traditional and ultra-processed foods (UPFs) in relation to child-feeding decisions. Your participation is voluntary and confidential, and your responses will be de-identified for analysis. The questionnaire will take about 10–15 minutes. You may skip any question and stop at any time.

Please continue only if you have read the consent information and agree to participate in this research study. If you are completing this questionnaire online, continuing indicates consent.

## Section 1. Demographic information

**1. Age of the mother (years):**

________________

**2. Emirate of residence:**

☐ Abu Dhabi ☐ Dubai ☐ Sharjah ☐ Ajman ☐ Umm Al Quwain ☐ Ras Al Khaimah ☐ Fujairah

**3. Educational level:**

☐ Primary ☐ Secondary ☐ University ☐ Postgraduate

**4. Occupation:**

☐ Housewife ☐ Teacher ☐ Nurse ☐ Doctor ☐ Businessperson ☐ Other: ________________

**5. Number of children:**

________________

**6. Age of youngest child (months):**

________________

## Section 2. Food photograph evaluation

*Instructions.* You will be shown a series of food images representing a variety of meals commonly available in the UAE, including traditional dishes and industrially prepared (ultra-processed) foods. If you have more than one child in this age range, please answer with your youngest child in mind.

*Definition provided to participants.* Ultra-processed foods (UPFs) are foods that are industrially produced and contain added sugars, fats, salt, flavorings, and preservatives. Examples include packaged snacks, sweetened beverages, instant noodles, and processed breakfast cereals.

**Rating items**

Each of the 12 food images listed below was presented individually and evaluated using the identical set of items shown here. For each food image, participants responded to the following:

**a. How would you rate the healthiness of this meal?**

☐ Very unhealthy ☐ Unhealthy ☐ Neutral ☐ Healthy ☐ Very healthy

**b. How likely are you to serve this food to your child?**

☐ Never ☐ Rarely ☐ Sometimes ☐ Often ☐ Always

**c. How convenient do you think this food is?**

☐ Very inconvenient ☐ Inconvenient ☐ Neutral ☐ Convenient ☐ Very convenient

**d. How tasty does this food look?**

☐ Very unappetizing ☐ Unappetizing ☐ Neutral ☐ Appetizing ☐ Very appetizing

**e. How processed do you think this food is?**

☐ Not at all processed ☐ Slightly processed ☐ Moderately processed ☐ Highly processed ☐ Extremely processed

**f. How culturally familiar is this food in your household?**

☐ Not at all ☐ Slightly ☐ Moderately ☐ Very ☐ Extremely

**g. Is this food suitable for your child's diet?**

☐ Yes ☐ No ☐ Not sure

**h. Why or why not? (open-ended)**

____________________________________________________________

Items (a)–(f) provided the six perception dimensions analyzed in this article. Items (g) and (h) were collected as part of the broader mixed-methods protocol and are not reported here.

**Food stimuli**

The 12 food images were presented in the alternating order shown below, with ultra-processed and traditional foods interleaved. Classification follows the NOVA system.

| **#** | **Food stimulus** | **Description** | **Classification** |
| --- | --- | --- | --- |
| 1 | Chicken nuggets with fries | Breaded chicken pieces served with fried potatoes | UPF |
| 2 | Grilled chicken with rice | Grilled chicken served with plain rice | Traditional |
| 3 | Chocolate cereal with flavored milk | Sweetened breakfast cereal with flavored milk | UPF |
| 4 | Khubz with labneh and dates | Flatbread with strained yogurt and dates | Traditional |
| 5 | Packaged snack cake + juice box | Pre-packaged snack cake with a boxed juice drink | UPF |
| 6 | Balaleet | Sweet vermicelli with cardamom, saffron, rose water, and eggs | Traditional |
| 7 | Instant noodles | Pre-cooked dried noodle product with seasoning | UPF |
| 8 | Vegetable salona | Traditional vegetable stew | Traditional |
| 9 | Sugar-sweetened beverages (SSBs) | Sweetened soft/juice drinks | UPF |
| 10 | Laban | Cultured buttermilk drink | Traditional |
| 11 | Chocolate bar with packaged cookies | Chocolate confectionery with packaged biscuits | UPF |
| 12 | Sago | Sago pearls with sugar, saffron, cardamom, and rose water | Traditional |

UPF, ultra-processed food. The corresponding food photographs presented to participants are not reproduced here; standardized images were drawn from A Photographic Atlas of Food Portions for the Emirate of Abu Dhabi and from research-team photographs of home-prepared dishes.

**Supplementary Table S5.** Threshold-specific odds ratios from the partial proportional-odds sensitivity model for willingness to serve foods, with the corresponding pooled odds ratios from the primary cumulative link mixed model (Table 2 of the main article).

| **Predictor** | **≥ 2 vs 1** | **≥ 3 vs ≤ 2** | **≥ 4 vs ≤ 3** | **5 vs ≤ 4** | **CLMM OR (Table 2)** |
| --- | --- | --- | --- | --- | --- |
| Food type (UPF vs traditional) | 4.49 (2.87–7.00) | 3.45 (2.54–4.68) | 1.45 (1.07–1.98) | 0.98 (0.59–1.61) | 1.23 (0.57–2.64) |
| Maternal age ≤29 y (vs 30–39 y) | 1.52 (1.08–2.15) | 0.99 (0.77–1.29) | 1.02 (0.79–1.31) | 0.93 (0.68–1.27) | 1.03 (0.79–1.34) |
| Maternal age ≥40 y (vs 30–39 y) | 1.27 (0.88–1.84) | 1.27 (0.97–1.67) | 1.01 (0.78–1.32) | 0.68 (0.50–0.92) | 1.01 (0.77–1.32) |
| Housewife (vs employed) | 1.19 (0.88–1.60) | 0.98 (0.79–1.22) | 0.94 (0.76–1.15) | 1.11 (0.87–1.41) | 1.07 (0.86–1.33) |
| Family size > 3 children (vs ≤ 3) | 1.85 (1.28–2.68) | 1.31 (1.01–1.71) | 1.30 (1.01–1.67) | 1.16 (0.85–1.57) | 1.44 (1.10–1.87) |
| Perceived healthiness | 1.47 (1.19–1.81) | 2.05 (1.76–2.38) | 1.85 (1.58–2.18) | 1.81 (1.39–2.36) | 1.64 (1.44–1.85) |
| Perceived convenience | 3.25 (2.62–4.02) | 2.21 (1.92–2.54) | 2.16 (1.83–2.55) | 2.30 (1.66–3.18) | 2.28 (2.03–2.56) |
| Perceived tastiness | 1.49 (1.33–1.68) | 1.33 (1.20–1.49) | 1.76 (1.54–2.02) | 2.28 (1.91–2.72) | 1.73 (1.59–1.88) |
| Cultural familiarity | 1.87 (1.65–2.12) | 2.46 (2.22–2.74) | 2.64 (2.35–2.97) | 2.46 (2.08–2.91) | 2.35 (2.16–2.55) |

OR, odds ratio; CI, confidence interval. Values are odds ratios with 95% confidence intervals. Estimates are from a partial proportional-odds cumulative link model (clm function, ordinal package, R version 4.5.1) fitted to the same 3,324 food-level observations from 277 mothers used in the primary analysis, with nominal (threshold-specific) effects specified for food type, maternal age group, occupation, family size, perceived healthiness, perceived convenience, perceived tastiness, and cultural familiarity. Predictors for which the proportional-odds assumption was supported (education, perceived processing, and all maternal belief and feeding-practice items) were retained as proportional-odds terms and are not shown. Each column gives the odds of responding above, rather than at or below, the indicated willingness-to-serve category; odds ratios were obtained by exponentiating the negative of the estimated nominal (threshold) coefficients. For Likert-type predictors, odds ratios correspond to a one-unit increase in the predictor. Because this model is a fixed-effects cumulative link model, it does not include the random intercepts for participant and food item used in the primary cumulative link mixed models; the estimates are therefore not directly comparable in magnitude with those in Table 2 of the main article and are presented to show the direction and variability of associations across response thresholds.
